# Mental health effects of the COVID-19 pandemic on children and young adults: empirical analysis of the past, present and the way forward

**DOI:** 10.1192/bjo.2023.617

**Published:** 2023-12-07

**Authors:** Mayank Gupta

**Affiliations:** Southwood Psychiatric Hospital, Pittsburgh, Pennsylvania, USA

**Keywords:** COVID-19, resilience, genetics, pandemic, mental health

## Abstract

The SARS-CoV-2 virus and its variants have had and are having serious implications for the mental health of the public. The critical limitations in the published literature for children, adolescents and young adults raise doubts about their clinical utility and overall generalisability. Amidst these gaps in knowledge, a twin study (Rimfeld et al) addresses several of these issues in relation to heritable individual differences and responses to environmental stressors. Besides calculating genetic correlation, the longitudinal study also compares symptoms at four different time points during the pandemic. These findings reflect a counterintuitive understanding of the role of resilience in the mental health of young adults in the UK. Unlike prior studies, this study focuses on methodological designs and underscores the applications of accurate statistical measures in observing these complex phenomena.

Scientific scepticism is a position that promotes suspension of judgement by reason until the proposition effects are examined by applying robust empirical methodologies. René Descartes’ (1596–1650) seminal work on doubt laid the foundations of modern research.^1^ To quote Descartes, ‘The first rule was never to accept anything as true unless I recognized it to be such: that is, carefully to avoid precipitation and prejudgment and to include nothing in my conclusions unless it presented itself so clearly and distinctly to my mind that there was no occasion to doubt it’.^1^ Likewise, influential British thinker Francis Bacon's (1561–1626) outstanding contributions during the scientific revolution were based on the core view that ‘whatever his mind seizes and dwells upon with particular satisfaction is to be held in suspicion’.^1^

## Categories of study conducted on the impacts of COVID-19 on mental health

The data from the SARS outbreak of 2003 was utilised in the faster determination of the SARS-CoV-2 virus via genomic sequencing, the development of its diagnostics and the implementation of robust strategies for quarantine and isolation.^2^ After the outbreak of the SARS-CoV-2 (COVID-19) pandemic in late 2019, stringent policies and measures were enforced to curb its spread.

These measures had an impact on the population's mental health and their effects were extensively studied worldwide.^3^ These studies were broadly divided into four categories: (a) effects of the lockdowns on mental health;^4^ (b) direct effects of the SARS-CoV-2 virus (and its variants)^5^ and the increased risk of incident mental health disorders in survivors of acute SARS-CoV-2 infection, including anxiety disorders, depressive disorders, stress and adjustment disorders, opioid use disorder, other (non-opioid) substance use disorders, neurocognitive decline and sleep disorders;^6^ (c) lasting symptoms post-infection (long COVID);^7^ and (d) worsening mental health emergencies secondary to the stress of the pandemic.^8^ Subsequently, in 2020 numerous published studies associated a range of mixed mental health effects with the pandemic lockdown. These indirect effects were linked to several perpetuating and precipitating factors, including restricted physical activity, heightened perceived risks and parenting stresses.^3^ A surge in mental health emergency visits was attributed to these factors, self-reports of symptoms of anxiety and depression in adolescents, and externalising disorders among college-aged youth.^9,10^

Another miscellaneous but highly important category includes studies specifically designed to test unanswered questions and serious limitations in the interpandemic literature. In this category, a prospective longitudinal observational study reported that anxiety and depressive symptoms peaked during the early stages of the first lockdown in England; there was a rapid decline over the subsequent 20-week period. Specific risk groups were also identified as female gender, individuals with lower educational attainment, lower income or pre-existing mental health conditions, and those living alone or with children.^11^ These types of study highlighted the role of confounding secondary stressors associated with variance in mental health outcomes.

During the initial phase of the pandemic, children, adolescents and young adults were considered less vulnerable to the direct effects of SARS-CoV-2, with very limited morbidity and mortality.^12^ It was not until late 2021 that both the indirect effects and the direct effects of the pandemic, including long COVID, were linked to negative mental health outcomes.^13^ However, further scrutiny through the application of the principles of scientific doubt and critical appraisal has brought to light methodological limitations, bringing under scrutiny the overall validity of these findings.^14^ There have been serious arguments about small effect sizes and omissions that contributed to a lack of generalisability.^15^ The key issues raised include the lack of a robust longitudinal study design that incorporates individual differences and measures many interacting variables.

## Children and young people and COVID-19: a twin study

To address some of these critical questions, Rimfeld et al designed and conducted a study of 4773 twins (mean age of 22 years) in the UK.^16^ In this sample, they measured and compared mental health symptoms at four distinct time points during the pandemic. Unlike prior studies, this study also accounted for the highly heritable differences in the response to changing environments (the pandemic) due to genetic variations between individuals.

Rimfeld et al measured mental health symptoms using self-reported questionnaires and the CoRonavIruS Health Impact Survey (CRISIS), which also assessed environmental factors germane to the pandemic.^16^ The genome-wide polygenic scores (GPS) estimated the variance in mental health measures at the four time points. The results were counterintuitive, and the study emphasised remarkable resilience among young adults which negated the effects of adversities during the pandemic. Resilience remains a complex construct with many definitions; however, in this context it is an individual's capacity to identify processes, including interpersonal social processes, that are statistically associated with stronger teams and relationships and, secondarily, the possibility of better outcomes.^17^

Although there was a decline in mental health during the initial phase of the lockdown, the participants exhibited no long-term negative effects. These results are contrary to previous findings; the authors emphasised that prior studies only focused on the statistical significance of the mean difference and not the effect size, which may have yielded negative effects. The computation of the standardised mean difference or effect size, often expressed as Cohen's *d*, is critical in providing a quantitative reflection of the magnitude of the phenomenon.^18^

The key findings include that individuals with pre-existing mental health conditions were more vulnerable to the effects of the pandemic. Even though the mental health symptoms got worse immediately after the lockdown, they came back to pre-pandemic levels during later stages. Another interesting finding indicated that the genetic correlation was 0.95 for mental health measures before and during the pandemic. This underscores that the genetic factors contributing to individual differences did not change during a pandemic.

Rimfeld et al have both highlighted critical gaps in the previous studies and provided many generalisable findings that could contribute to policy-making and healthcare management. First, equitable study designs are crucial for empirically measuring true estimates of any effect. The difference in the results as compared with the previous studies was due to reasons such as omitted variables bias, small effect size, shorter duration and inter-participant variability. Second, individuals with pre-existing psychiatric illnesses remain the most vulnerable populations, and policymakers need to prioritise making mental healthcare services easily accessible to them. Third, the positive effects of an individual's strengths and resilience factors were underestimated and even omitted in previous studies. Future studies to identify the role of resilience in the context of pandemic-related adversities will help implement population-based measures to better stratify healthcare resources. However, it is important to note that the Rimfeld et al study has many limitations, including the implicit bias of using self-reported questionnaires among predominately White educated young adults in the UK.

## The way forward

In the past 2 years, more data have come up that suggest the presence of long COVID symptoms in children, adolescents and young adults.^19^ Similarly, concerns about the validity of these findings are questioned since many with negative test results (reverse transcription polymerase chain reaction) had similar symptomatology of long COVID as those with positive test results.^20^ The self-limiting long COVID symptoms often remit in 6 weeks; however, long COVID continues to burden the healthcare system and adds to the conundrum.^21^ The role of interacting variables such as immunological factors and psychological stress, and their relationship with response to SARS-CoV-2, remains an area of future research. There are many variants of SARS-CoV-2 and differences in mental health sequelae among vaccinated and unvaccinated would inspire a better understanding of variability in responses.

There is compelling evidence from cross-country comparative studies about the effectiveness of non-pharmaceutical interventions (NPIs) to mitigate the spread of COVID-19. Although there was a large effect of the timely closing of both schools and universities, the stay-at-home order had a small effect when a country had already closed its academic institutions and non-essential businesses and banned gatherings.^22^ These lessons from the chronology of cross-country implementation of NPI have yielded the roadmap for future strategies and research.

There was an urgent need in 2020 to recognise and understand the mental health impact of the SARS-CoV-2 virus. There were calls for papers from the editorial teams of several journals and literature was freely available without access fees. During the initial phase, many studies provided a broad trend, with many serious gaps in the methodologies. More detailed analysis of these data and better-designed studies provided different findings in subsequent years. In times of misinformation, when the wide reach of unmonitored social media has contributed to vaccine hesitancy, it is imperative to get the messaging right.^23^ The research and clinical community must adhere to the principles of scientific doubt, with openness about the inherent flaws in study designs, implicit bias and gaps in the level of evidence generated.

## Data Availability

Data availability is not applicable to this article as no new data were created or analysed in this study.

## References

[1] Ijsseling S. Francis Bacon, René Descartes and the new science. In Rhetoric and Philosophy in Conflict: An Historical Survey (ed S Ijsseling): 60–70. Springer, 1976.

[2] Yang Y, Peng F, Wang R, Guan K, Jiang T, Xu G, et al. The deadly coronaviruses: the 2003 SARS pandemic and the 2020 novel coronavirus epidemic in China. J Autoimmun 2020; 109: 102434. 32143990 10.1016/j.jaut.2020.102434 PMC7126544

[3] Meade J. Mental health effects of the COVID-19 pandemic on children and adolescents. Pediatr Clin North Am 2021; 68: 945–59. 34538305 10.1016/j.pcl.2021.05.003 PMC8445752

[4] Jia R, Ayling K, Chalder T, Massey A, Broadbent E, Coupland C, et al. Mental health in the UK during the COVID-19 pandemic: cross-sectional analyses from a community cohort study. BMJ Open 2020; 10(9): e040620. 10.1136/bmjopen-2020-040620 PMC7493070 32933965

[5] Toubiana J, Poirault C, Corsia A, Bajolle F, Fourgeaud J, Angoulvant F, et al. Kawasaki-like multisystem inflammatory syndrome in children during the COVID-19 pandemic in Paris, France: prospective observational study. BMJ 2020; 369: m2094. 32493739 10.1136/bmj.m2094 PMC7500538

[6] Xie Y, Xu E, Al-Aly Z. Risks of mental health outcomes in people with COVID-19: cohort study. BMJ 2022; 376: e068993. 35172971 10.1136/bmj-2021-068993 PMC8847881

[7] Gupta M, Gupta N, Esang M. Long COVID in children and adolescents. Prim Care Companion CNS Disord 2022; 24(2): 21r03218. 10.4088/PCC.21r03218 35486940

[8] Yard E. Emergency department visits for suspected suicide attempts among persons aged 12–25 years before and during the COVID-19 pandemic – United States, January 2019–May 2021. MMWR Morb Mortal Wkly Rep 2021; 70: 888–94. 34138833 10.15585/mmwr.mm7024e1 PMC8220953

[9] Feroz AS, Ali NA, Ali NA, Feroz R, Meghani SN, Saleem S. Impact of the COVID-19 pandemic on mental health and well-being of communities: an exploratory qualitative study protocol. BMJ Open 2020; 10(12): e041641. 10.1136/bmjopen-2020-041641 PMC7780420 33384393

[10] Varma P, Junge M, Meaklim H, Jackson ML. Younger people are more vulnerable to stress, anxiety and depression during COVID-19 pandemic: a global cross-sectional survey. Prog Neuropsychopharmacol Biol Psychiatry 2021; 109: 110236. 33373680 10.1016/j.pnpbp.2020.110236 PMC7834119

[11] Fancourt D, Steptoe A, Bu F. Trajectories of anxiety and depressive symptoms during enforced isolation due to COVID-19 in England: a longitudinal observational study. Lancet Psychiatry 2021; 8: 141–9. 33308420 10.1016/S2215-0366(20)30482-X PMC7820109

[12] Zimmermann P, Curtis N. Why is COVID-19 less severe in children? A review of the proposed mechanisms underlying the age-related difference in severity of SARS-CoV-2 infections. Arch Dis Child [Epub ahead of print] 1 Dec 2020. Available from: 10.1136/archdischild-2020-320338. 33262177

[13] Cui J, Lu J, Weng Y, Yi GY, He W. COVID-19 impact on mental health. BMC Med Res Methodol 2022; 22(1): 15. 35026998 10.1186/s12874-021-01411-w PMC8758244

[14] Radtke T, Ulyte A, Puhan MA, Kriemler S. Long-term symptoms after SARS-CoV-2 infection in children and adolescents. JAMA 2021; 326: 869–71. 34264266 10.1001/jama.2021.11880 PMC8283661

[15] Nieto I, Navas JF, Vázquez C. The quality of research on mental health related to the COVID-19 pandemic: a note of caution after a systematic review. Brain Behav Immunity Health 2020; 7: 100123. 10.1016/j.bbih.2020.100123 PMC7395946 32835299

[16] Rimfeld K, Malanchini M, Arathimos R, Gidziela A, Pain O, McMillan A, et al. The consequences of a year of the COVID-19 pandemic for the mental health of young adult twins in England and Wales. BJPsych Open 2022; 8(4): e129. 35860899 10.1192/bjo.2022.506 PMC9304950

[17] Southwick SM, Bonanno GA, Masten AS, Panter-Brick C, Yehuda R. Resilience definitions, theory, and challenges: interdisciplinary perspectives. Eur J Psychotraumatol 2014; 5: 25338. 10.3402/ejpt.v5.25338 PMC4185134 25317257

[18] Kelley K, Preacher KJ. On effect size. Psychol Methods 2012; 17: 137–52. 22545595 10.1037/a0028086

[19] Stephenson T, Pinto Pereira SM, Shafran R, de Stavola BL, Rojas N, McOwat K, et al. Physical and mental health 3 months after SARS-CoV-2 infection (long COVID) among adolescents in England (CLoCk): a national matched cohort study. Lancet Child Adolesc Health 2022; 6: 230–9. 35143770 10.1016/S2352-4642(22)00022-0 PMC8820961

[20] Molteni E, Sudre CH, Canas LS, Bhopal SS, Hughes RC, Antonelli M, et al. Illness duration and symptom profile in symptomatic UK school-aged children tested for SARS-CoV-2. Lancet Child Adolesc Health 2021; 5: 708–18. 34358472 10.1016/S2352-4642(21)00198-X PMC8443448

[21] Gurdasani D, Akrami A, Bradley VC, Costello A, Greenhalgh T, Flaxman S, et al. Long COVID in children. Lancet Child Adolesc Health 2022; 6(1): e2. 34921807 10.1016/S2352-4642(21)00342-4 PMC8673872

[22] Brauner JM, Mindermann S, Sharma M, Johnston D, Salvatier J, Gavenčiak T, et al. Inferring the effectiveness of government interventions against COVID-19. Science 2021; 371(6531): eabd9338. 33323424 10.1126/science.abd9338 PMC7877495

[23] Garett R, Young SD. Online misinformation and vaccine hesitancy. Transl Behav Med 2021; 11: 2194–9. 34529080 10.1093/tbm/ibab128 PMC8515268

